# Application of Cyclized Polyacrylonitrile for Ultrafiltration Membrane Fouling Mitigation

**DOI:** 10.3390/membranes12050489

**Published:** 2022-04-30

**Authors:** Alexandra Pulyalina, Nadezhda Tian, Anna Senchukova, Ilya Faykov, Maria Ryabikova, Alexander Novikov, Natalia Saprykina, Galina Polotskaya

**Affiliations:** 1 Institute of Chemistry, Saint Petersburg State University, 198504 Saint Petersburg, Russia; tyan-nadezhda91@yandex.ru (N.T.); st024726@student.spbu.ru (A.S.); st022544@student.spbu.ru (I.F.); st069152@student.spbu.ru (M.R.); a.s.novikov@spbu.ru (A.N.); polotskaya@hq.macro.ru (G.P.); 2 Institute of Macromolecular Compounds, Russian Academy of Sciences, 199004 Saint Petersburg, Russia; saprykina@hq.macro.ru

**Keywords:** ultrafiltration, polymer composites, cyclized polyacrylonitrile, poly(amide-imide)

## Abstract

In this study, novel composites were produced by blending partially cyclized polyacrylonitrile (*c*PAN) and poly(amide-imide) (PAI) in N-methylpyrrolidone in order to fabricate asymmetric membranes via phase inversion method. The compatibility of PAI and *c*PAN through possible intermolecular interaction was examined by quantum chemical calculations. The composite membranes were characterized by FTIR, SEM, contact angle measurements, etc. A considerable reduction in the contact angles of water and ethylene glycol (EG) was observed after adding *c*PAN to the PAI membrane, which is evidence of improved membrane hydrophilicity. Membrane transport properties were investigated in ultrafiltration tests by measuring the pure water flux, rejection of proteins, and flux recovery ratio (*FRR*). The best properties were found for the membrane containing 5 wt% *c*PAN; an increase in BSA rejection and a remarkable increase in *FRR* were observed, which can be explained by the hydrophilization of the membrane surface provided by the presence of *c*PAN.

## 1. Introduction

At present, membrane methods are widely applied for the separation of liquid media [1,2,3,4,5]. Ultrafiltration (UF) is one of the most common membrane methods, which is widely used for wastewater treatment in food and biopharmaceutical industries due to its low energy consumption, ease of operation, and high efficiency [6,7]. UF allows for improved quality of life by purifying water, which is the basis of all living organisms, of dissolved organic macromolecules, suspended particles, viruses, etc. [8,9]. An ideal UF membrane has high permeability and rejection, as well as anti-fouling properties. However, it should be recognized that the performance and anti-fouling properties of known polymeric membranes are rather low due to their hydrophobic nature, which leads to membrane fouling [10,11]. Membrane contaminants such as proteins, microorganisms, and colloidal solids can deposit on a membrane surface and block pores, which has an adverse effect on membrane performance as a result of reduced fluxes, changing membrane selectivity, increased operating costs, and shortened membrane life cycles [12,13]. Therefore, a highly relevant step is to modify UF membranes to overcome membrane fouling and enhance water fluxes [14,15,16]. Common methods include coating and grafting on the membrane surface [17,18]. However, the blending method is simpler and more versatile for controlling the membrane structure, properties, and filtration efficiency [19]. It should be recognized that the blending method often faces the issue of poor compatibility of blended polymers, which can lead to a rough membrane surface and defect formation. Polymers with similar molecular structure generally exhibit good compatibility, and their blending leads to the improvement of membrane operational characteristics [20,21].

**Figure 1:**
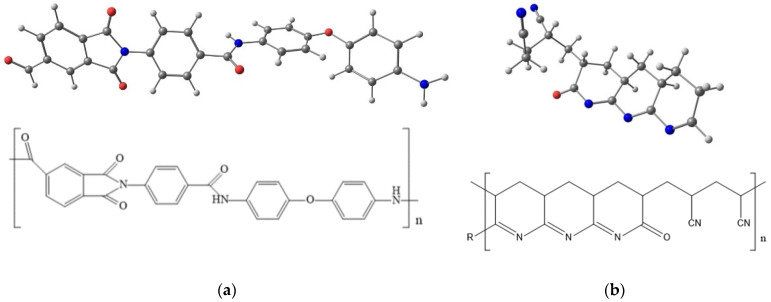
Molecular structures of (**a**) PAI and (**b**) *c*PAN.

Membranes based on aromatic poly(amide-imides) are being actively developed and researched [22,23,24,25,26]. In this work, we study membranes based on poly[(4,4′-bisamide)-oxydiphenylene-N-(*p*-phenylene)-4-phthalimide] (PAI), i.e., poly(amide-imide) (Figure 1), which has been established as a membrane material in gas separation [27,28] and pervaporation [29] processes. 

This PAI belongs to the class of polyheteroarylenes, and its high thermal, mechanical, and transport properties are determined by the presence of the amide and imide functional groups in the monomer unit. A.N. Cherkasov et al. produced a defect-free, asymmetric flat-sheet PAI membrane [30].

The transport properties of UF membranes are significantly affected by the introduction of various additives, including polymeric additive [31]. In this work, PAI is modified by an additive of partially cyclized polyacrylonitrile (*c*PAN), in which the parts of nitrile groups of *c*PAN were converted into the cyclic sequences containing polyconjugated bonds of the imine and carbonyl groups as a result of chemical treatment with organometallic compounds [32,33]. *c*PAN is not a flexible polymer and cannot form films on its own. However, it is resistant to most organic solvents, swells and dissolves only in amide solvents (in particular, *N*-methylpyrrolidone), and has high thermal and chemical stability [34]. *c*PAN has been used as a polymer additive for obtaining UF polyimide membranes from polyamic acid, where it also acts as an imidization catalyst [35].

The aim of this work is to select the conditions for obtaining UF asymmetric PAI membranes with increased permeability, to create an ultraporous asymmetric membrane from a blend of PAI and *c*PAN, and to study the effect of *c*PAN on the structure and transport properties of membranes based on PAI.

## 2. Materials and Methods

### 2.1. Materials

PAI was synthesized by low-temperature polycondensation in *N*-methylpyrrolidone (NMP) as described in [36].

Polyacrylonitrile (PAN) was synthesized by anionic polymerization; *c*PAN was obtained from PAN by treatment with lithium *tert*-butoxide in dimethyl formamide in an inert gas atmosphere at a temperature ≥0 °C [37]. The process was terminated by adding acetic acid to the solution; then, the reaction mixture was filtered, and the polymer was isolated by precipitation into water. After washing with water, *c*PAN powder was dried in air.

The structures of PAI and *c*PAN are shown in Figure 1.

### 2.2. Preparation of Membranes

Asymmetric membranes were obtained by the phase inversion technique. To prepare a polymer blend in a casting solution, calculated amounts of *c*PAN powder (5, 10, or 15 wt%) were added to a PAI solution in *N-*methylpyrrolidone (NMP) under thorough stirring.

Asymmetric membranes were obtained by casting a 12 wt% solution of PAI or PAI-*c*PAN in NMP onto a glass plate using a casting knife with a gap of 0.3 mm. Then, the glass plate with the polymer solution was immersed into a coagulating bath at room temperature. Water or a water/ethanol mixture (60/40 *w*/*w*) was used as a coagulant. The formed membrane was kept in the coagulating bath for ~3 h. The membrane was then washed with water, ethanol, and hexane and dried. The composite membranes containing 5, 10, and 15 wt% of *c*PAN additive are denoted as PAI-5, PAI-10, and PAI-15, respectively, in this work.

### 2.3. Computational Details

Full geometry optimization of all model structures was carried out at the PM6 level of theory with the help of the Gaussian-09 program package (Gaussian, Inc., Wallingford CT, USA) [38]. No symmetry restrictions were applied during the geometry optimization procedure. The Hessian matrices were calculated for all optimized model structures to prove the location of correct minima on the potential energy surface (no imaginary frequencies were found in any case). The thermodynamic parameters were calculated at 298.15 K and 1.00 atm (Table S1, Supplementary Materials). The Cartesian atomic coordinates for all optimized equilibrium model structures are presented in Supplementary Materials as XYZ files.

### 2.4. Characterization

The presence of functional groups and their intensities were analyzed via a Bruker Tensor 27 FTIR spectrometer (Bruker Daltonics, Bremen, Germany) with a resolution of 1 cm^−1^ in the range of 500–4000 cm^−1^ at 25 °C.

Membrane morphology was studied by scanning electron microscopy (SEM) using a Zeiss SUPRA 55VP (Carl Zeiss, Oberkochen, Germany) microscope. To prepare a sample for SEM, the dried membrane was cracked in liquid nitrogen and then coated with a 20 nm thick platinum layer using a Quorum 150 cathode-sputtering installation (Quorum Technologies Ltd., Lewes, UK).

Contact angles of liquids on membrane surfaces were measured via the sessile drop method as described in [39] at ambient temperature and atmospheric pressure. Liquids under study were water and ethylene glycol, with a surface tension equal to 72.4 mN/m and 47.7 mN/m, respectively.

Ultrafiltration experiments were carried out in an FM-01 dead-end stirred cell at ambient temperature; the membrane diameter was 29 mm, and the initial filtration volume was 10 mL [40]. A transmembrane pressure of ~1.6 bar was created by supplying a nitrogen flow. The amount of permeate (filtrate) was determined by the weight method.

The data of UF experiments were used to calculate the transport properties of the membranes. The flux through the membrane, *J* (m·h^−1^·bar^−1^), was calculated as:

(1) $$J=\frac{V}{t\cdot S\cdot P}$$

where *V* is the volume of the permeate (m^3^), *t* is the filtration time (h), *S* is the membrane surface area (m^2^), and *P* is the transmembrane pressure (bar).

Separation efficiency of the membranes was determined in UF experiments using 1 g/L aqueous solutions of various proteins (Table 1) according to the technique described in [41].

The protein concentration in the feed and permeate was determined using a PE-5400UF spectrophotometer. The measurements were carried out at a wavelength of 280 nm.

The rejection (*R*) was calculated by the following equation [42]:

(2) $$R=\left(1-\frac{C_{p}}{C_{0}}\right)\;\cdot 100\%,$$

where *C_p_* is the protein concentration in the filtrate, and *C*_0_ is the protein concentration in the feed (g/L).

The flux recovery ratio (*FRR*) was calculated using the following equation:

(3) $$\mathrm{FRR}=\frac{J_{0t}}{J_{0}},$$

where *J*_0_ is the pure water flux through the membrane, and *J*_0__t_ is the pure water flux after ultrafiltration of the protein solution at the same pressure.

## 3. Results and Discussion

### 3.1. Transport Properties of Poly(Amide-Imide) (PAI)

The membranes studied in this work were prepared from poly[(4,4′-bisamide)oxydiphenylene-N-(p-phenylene)-4-phtalimide] (PAI) (Figure 1); the main physical and mechanical properties of this polymer are given in Table 2 [36]. The high density, high glass transition temperature, and high level of mechanical properties of this polymer should contribute to the formation of mechanically strong, thermally and chemically resistant membranes from this polymer.

It is known that the conditions of formation of asymmetric membranes strongly affect their morphology and transport parameters [43]. Thus, one successful method for increasing the productivity of membranes is to vary the composition of the coagulant in the precipitation bath. The conventional and most accessible coagulant for polyheteroarylene membranes is water. In this work, another coagulant that combines soft and hard precipitants was studied, namely water and ethanol in a ratio of 60/40 wt%.

**Figure 2:**
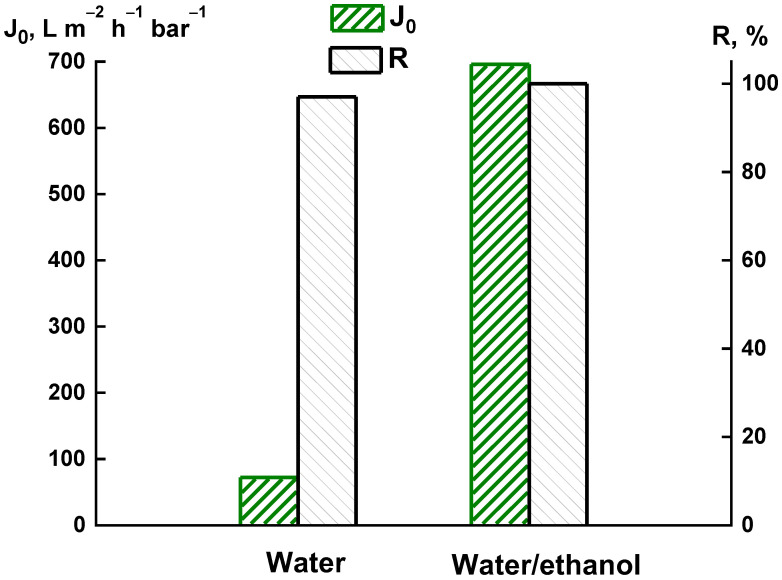
Dependence of pure water flux (*J*_0_) and BSA rejection (*R*) on the composition of coagulation bath.

Figure 2 shows the dependence of pure water flux and rejection of bovine serum albumin (BSA) on the composition of the coagulation bath. Changing the composition of the bath significantly affects the transport properties of the membranes. The pure water flux increased by nine times when using the water/ethanol mixture (60/40 wt%) as a coagulant. In addition, when using the water/ethanol mixture (60/40 wt%) as a coagulant, the PAI membrane demonstrated the maximum rejection (100%), whereas this value for the membrane prepared in water bath was equal to 97%. In our opinion, the improvement of PAI membrane properties is connected with the fact that the water/ethanol mixture (60/40 wt%) is a softer coagulant than pure water [42].

**Figure 3:**
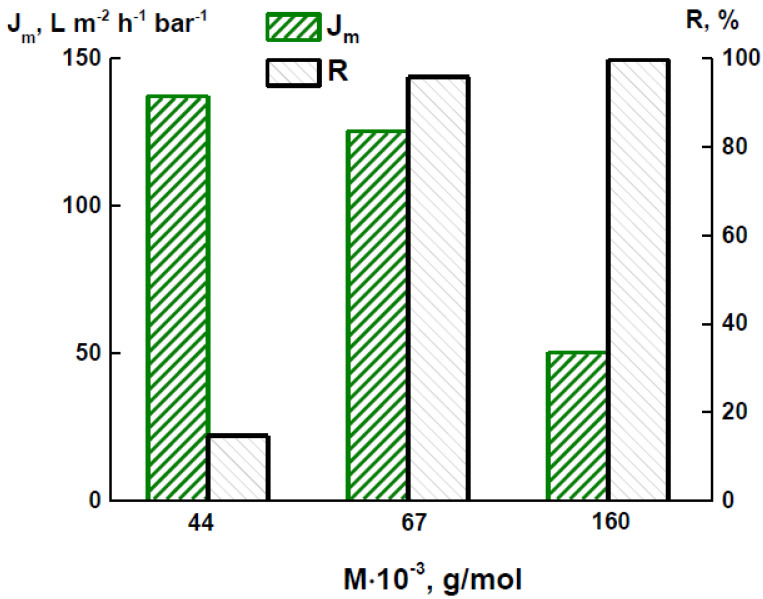
Dependences of the flux of aqueous protein solutions (*J_m_*) and rejection of proteins (*R*) on their molecular weights for the PAI membrane.

All the membranes further studied were prepared from a 12 wt% PAI solution in NMP using the water/ethanol mixture (60/40 wt%) as a coagulant. To study the transport properties of the PAI membranes in detail, UF experiments were carried out with the aqueous solutions of proteins with different molecular weights: ovalbumin (44,000 g/mol), bovine serum albumin (67,000), and γ-globulin (160,000). Figure 3 shows the dependences of the flux of aqueous protein solutions and rejection of proteins on their molecular weights. With an increase in the molecular weight of a protein, a decrease in the flux and an increase in the rejection were observed. Ovalbumin (as the smallest protein molecule) showed the highest permeability but the lowest rejection. When filtering the solutions of bovine serum albumin and γ-globulin, the PAI membrane demonstrated a rejection rate close to 100%.

### 3.2. Membrane Structure and Characterization

The transport properties of UF membranes are significantly affected by the introduction of various additives, including polymeric additive, in the membrane composition [44,45]. In this work, partially cyclized polyacrylonitrile (*c*PAN) was used as an additive to PAI. During membrane formation from PAI/*c*PAN composites, it was shown that the introduction of up to 15 wt% of *c*PAN led to the formation of single-phase solutions, from which asymmetric membranes could be formed. The composite membranes containing 5, 10, and 15 wt% *c*PAN additives in PAI are denoted as PAI-5, PAI-10, and PAI-15, respectively.

**Figure 4:**
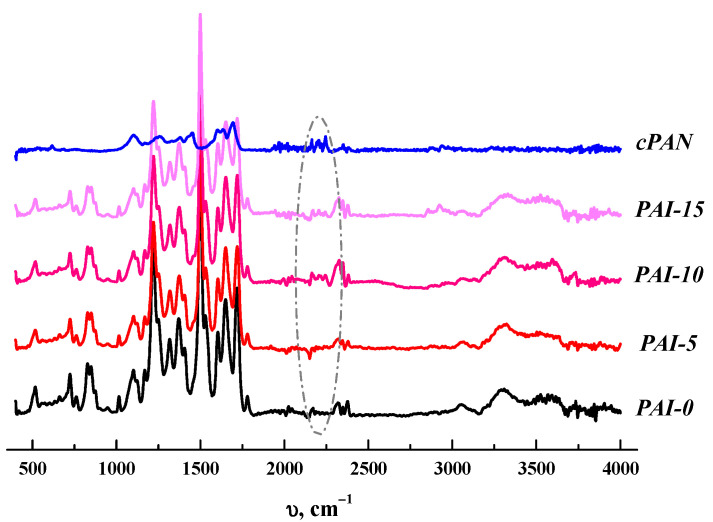
ATR-FTIR spectra of *c*PAN, PAI-0, PAI-5, PAI-10, and PAI-15.

Membrane composition was studied by FTIR spectrometry. Figure 4 shows the spectra of PAI, *c*PAN, and their composites. The vibration band corresponding to the nitrile group of *c*PAN is present at 2245 cm^−1^, and the vibration band assigned to C–H stretching vibrations of *c*PAN appears at about 3000 cm^−1^. The bands characteristic of pure PAI are as follows: the band corresponding to N-C=O stretching vibrations (1603 cm^−1^), the band assigned to C=O stretching vibrations of the amide group (1651 cm^−1^), as well as the bands corresponding to symmetric and asymmetric C-O-C stretching vibrations (1015 and 1220 cm^−1^, respectively). Furthermore, the bands corresponding to symmetric and asymmetric C=O stretching vibrations also appear in the spectrum of pure PAI. As can be seen in Figure 4, the bands characteristic of *c*PAN also appear in the spectra of the composite membranes. In particular, the band assigned to the nitrile group appears at about 2250 cm^−1^ for PAI-5, PAI-10, and PAI-15, which confirms the presence of *c*PAN in the structure of the composites. This band is the most intensive in the case of PAI-10 and PAI-15.

**Figure 5:**
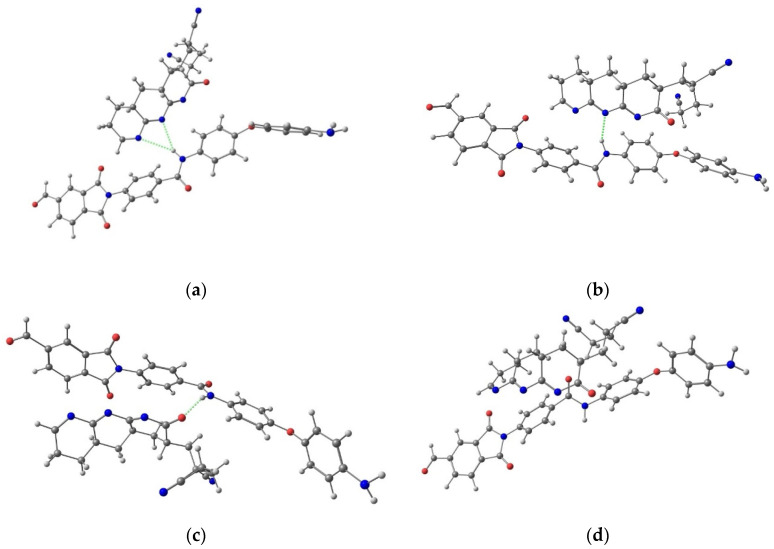
Supramolecular associates (**a**) A, (**b**) B, (**c**) C, and (**d**) D.

It is known that the presence of intermolecular interactions contributes to better compatibility of polymers [35]. The nature of interactions between the two polymers used as components of the composites in this work was estimated using quantum calculations. The results of quantum chemical calculations (Table 3 and Table S1) suggest the existence of four minima on the potential energy surface for hypothetical supramolecular adducts formed by the model structures of PAI and *c*PAN: adduct A, with bifurcated N–H···N intermolecular hydrogen bonds between the carboxamide group of PAI and two N atoms of the imine moieties in *c*PAN; adduct B, with N–H···N intermolecular hydrogen bonds between the carboxamide group of PAI and N atoms of the central imine moiety in *c*PAN; adduct C, featuring N–H···O intermolecular hydrogen bonds between the carboxamide group of PAI and O atoms of *c*PAN; and adduct D, bonded by multiple weak, noncovalent contacts involving C–H and Ph moieties but without a noticeable participation of N and O atoms from *c*PAN (Figure 5). The hypothetical supramolecular association process of PAI + *c*PAN → D is the most thermodynamically favorable (exothermic by 16.4 kcal/mol and exergonic by 0.9 kcal/mol).

**Figure 6:**
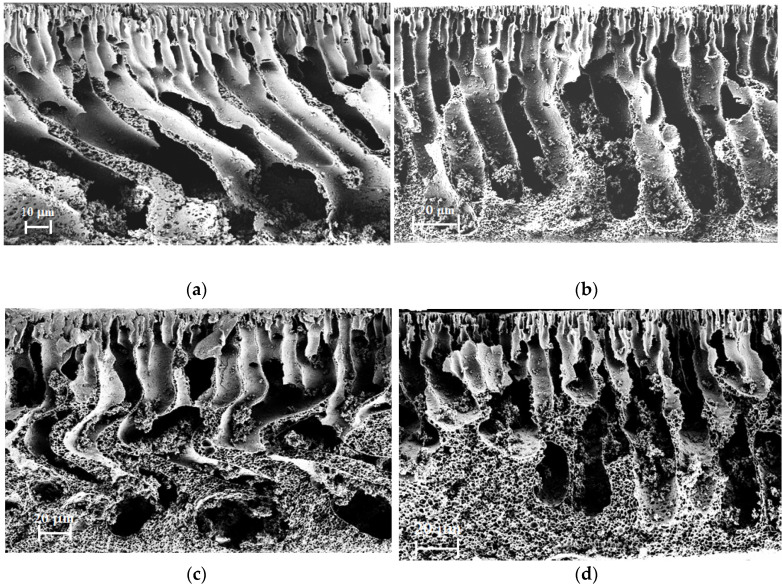
SEM micrographs of cross sections of the (**a**) PAI-0, (**b**) PAI-5, (**c**) PAI-10, and (**d**) PAI-15 membranes.

Scanning electron microscopy (SEM) was used to study the membrane morphology. Figure 6 shows the cross-section micrographs for the PAI-0, PAI-5, PAI-10, and PAI-15 membranes. The cross sections of all the membranes have an anisotropic structure consisting of a thin top layer and a porous substrate. The micrograph of the PAI-0 membrane demonstrates a finger-like structure throughout the thickness of the cross section. For PAI-5, PAI-10, and PAI-15 composite membranes, the structure of the cross section changes to a combined porous structure. Figure 6b–d shows that the *c*PAN addition leads to the formation of a spongy structure towards the bottom surface, along with a finger-like structure. The content of the spongy structure increases with increasing *c*PAN content in the composite.

We assume that the change in the membrane morphology after adding *c*PAN is due to the formation of a supramolecular structure as a result of interactions between the *c*PAN and PAI molecules. This assumption is supported by the results of quantum chemical calculations discussed above (Table 3 and Figure 5). The above interactions lead to the formation of PAI-*c*PAN associates and microheterogeneity, which results in a more porous structure of the PAI-*c*PAN composite membranes.

The nature of the change in the surface properties of the PAI membrane modified with *c*PAN was estimated by measuring the contact angles of two liquids (water and ethylene glycol) on the membrane surfaces (Table 4). The water contact angle of pure PAI is 56°, and it decreases with increasing *c*PAN content in the composite membrane. The ethylene glycol contact angle of the membrane surface has the same tendency to decrease after adding *c*PAN. Thus, the introduction of *c*PAN contributes to hydrophilization of the PAI membrane. This phenomenon may be explained using the data of quantum chemical calculations. Intermolecular interactions between the *c*PAN and PAI molecules result in the formation of supramolecular structures (adducts), where hydrophobic Ph moieties are localized inside an associate, whereas more hydrophilic fragments are situated on the surface.

### 3.3. Transport Properties of the Polymer Composites (PAI/cPAN)

**Figure 7:**
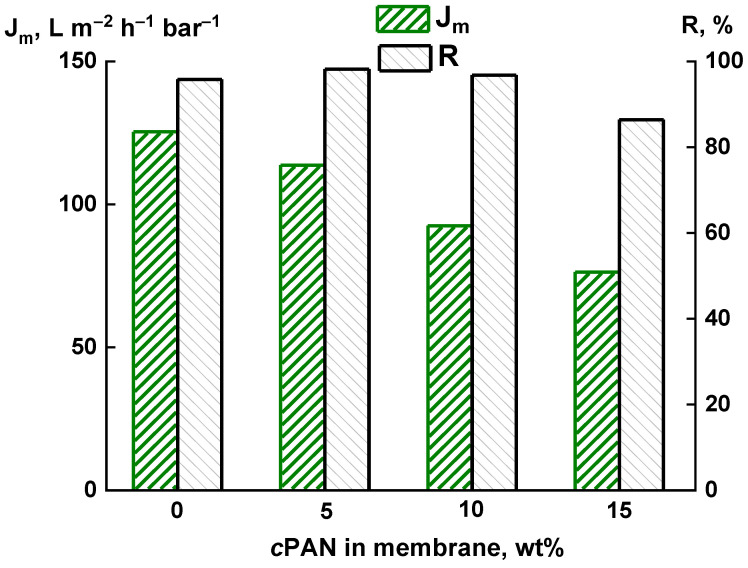
Flux of aqueous BSA (*J_m_*) and BSA rejection (*R*) vs. *c*PAN content in the membrane.

The transport properties of the PAI/*c*PAN membranes were studied in UF experiments with an aqueous solution of bovine serum albumin. Figure 7 shows the dependence of aqueous BSA flux and BSA rejection on the *c*PAN content in the membrane. The introduction of *c*PAN leads to a decrease in permeability. For the PAI-5 membrane, the permeability of the aqueous BSA slightly decreases compared to the PAI membrane. At the same time, high values of BSA rejection are observed for all the membranes. For the PAI-5 membrane, the BSA rejection exceeds 98 wt%.

**Figure 8:**
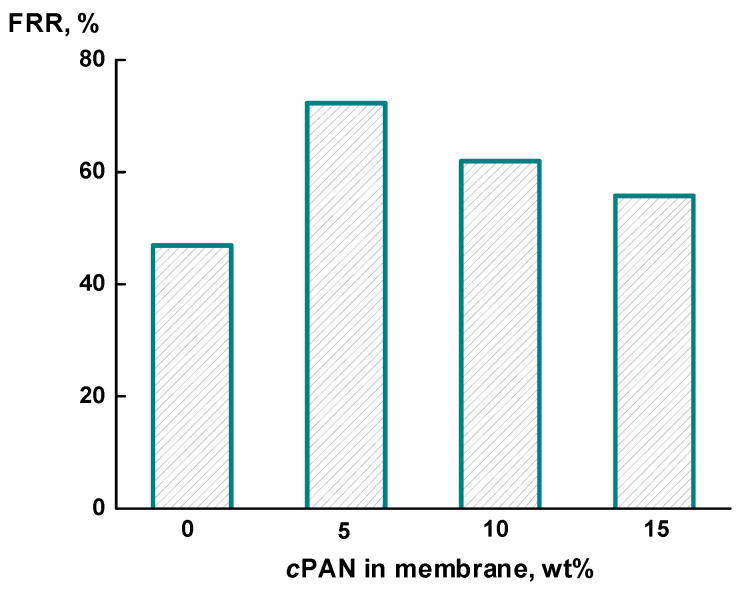
Dependence of flux recovery ratio on their molecular weights for PAI-*c*PAN membranes.

It is particularly noteworthy that *c*PAN additives increase the *FRR*. Figure 8 demonstrates that the introduction of 5 wt% *c*PAN contributes to a significant increase in the *FRR* up to 72% (compared to 46% for pure PAI). This fact can be explained by the hydrophilization of the membrane and pore surfaces after the addition of *c*PAN, which prevents fouling of the composite membrane [15,18]. A further increase in the *c*PAN content in the membrane does not have such a pronounced effect on the *FRR*, although the *FRR* value for all the composites exceeds that for the PAI membrane.

A significant increase in the *FRR* for PAI-5 as compared with PAI can be associated with a sharp decrease in the water contact angle and an increase in hydrophilicity, which results in an increase in the *FRR*. As compared with PAI-10 and PAI-15, the predominant finger-like structure of the porous substrate of PAI-5 is more accessible for regeneration after the filtration of protein solutions. Thus, the enhanced *FRR* of PAI-5 is associated with hydrophilization and a change in the structure and shape of the microporous substrate [15,18].

**Figure 9:**
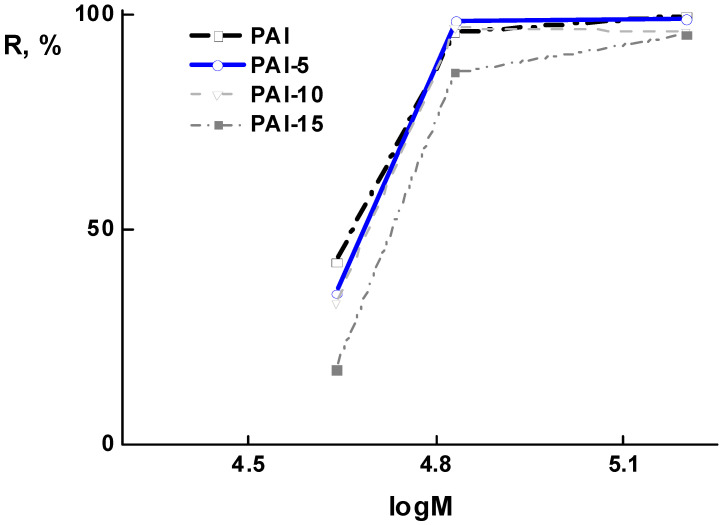
Dependence of proteins’ rejection on their molecular weights for PAI-*c*PAN membranes.

To determine the value of the molecular weight cut-off (*MWCO*) of the studied membranes, UF experiments were carried out with aqueous solutions of ovalbumin, bovine albumin, and γ-globulin. Figure 9 shows the dependence of rejection on the protein molecular weight for all membranes under study. The data shown in Figure 9 allowed us to determine the value of the *MWCO*, which corresponds to the weight of a protein rejected by 90% [32]. The PAI-5 and PAI-10 membranes are typical ultrafilters, with an *MWCO* equal to 60 × 10^3^ g/mol. The PAI-15 membrane shows a rather low efficiency.

Thus, it was found that the composite containing 5 wt% *c*PAN exhibits the optimal transport characteristics. The introduction of *c*PAN leads to hydrophilization of the membrane surface and porous substrate, which prevents membrane fouling.

## 4. Conclusions

In this work, novel composites were prepared by blending PAI and *c*PAN in casting solutions in order to fabricate asymmetric membranes via phase inversion method. A water/ethanol (60/40 wt%) mixture was used as a coagulation medium. The SEM studies shows the formation of an anisotropic structure consisting of a thin top layer and a porous substrate. The porous substrate of pure the PAI membrane has a finger-like structure. The introduction of *c*PAN led to the formation of a spongy structure near the bottom surface. The content of the spongy structure increases with *c*PAN content in the composite.

The compatibility of PAI and *c*PAN through possible intermolecular interaction was investigated by FTIR and quantum chemical calculations. It was suggested that hypothetical thermodynamically favorable supramolecular adducts could be formed. Measurements of water and ethylene glycol contact angles showed that the contact angle values for *c*PAN/PAI composites containing 5, 10, and 15 wt% of *c*PAN were lower than those of the pure PAI matrix, which indicates enhanced membrane hydrophilicity.

Membrane transport properties were investigated in UF tests by measuring the pure water flux, protein rejection, and flux recovery ratio. The best properties were found for the membrane containing 5 wt% *c*PAN, characterized by an increase in BSA rejection up to 98%. Furthermore, the addition of 5 wt% *c*PAN contributes to a significant increase in the flux recovery ratio up to 72%, which can be explained by hydrophilization of the membrane and pore surface. UF tests with proteins of different molecular weight showed that the composite membranes developed in this work are typical ultrafiltration membranes. The molecular weight cut-off is about 60 × 10^3^ g/mol for the PAI-5 and PAI-10 membranes.

It should be noted that the use of *c*PAN as a modifier is promising in terms of increasing the flux recovery ratio. The relatively high *FRR* of the composite membranes (compared to that of pure PAI) is an important advantage because this factor can facilitate the process of membrane regeneration and purification and reduce the loss of target components.

## Figures and Tables

**Table 1:** Molecular weights (*M*) and Stokes radii (*r_S_*) of protein molecules.

Protein	*M* 10^−3^, g/mol	*r_S_*, Ǻ
Ovalbumin	44.0	28.6
Bovine serum albumin	67.0	34.0
γ-globulin	160.0	46.5

**Table 2:** Physical and mechanical properties of PAI [36].

Property	Unit	Value
Molecular weight	g·mol^−1^	60,000
Density	g·cm^−3^	1.50
Glass transition temperature	°C	230
Break stress	MPa	125
Ultimate deformation	%	53

**Table 3:** Calculated values of enthalpies and Gibbs free energies of reaction (ΔH and ΔG) for various hypothetical supramolecular association processes (in kcal/mol).

Supramolecular Association Process	ΔH	ΔG
PAI + *c*PAN → A	−8.0	3.0
PAI + *c*PAN → B	−8.3	5.7
PAI + *c*PAN → C	−10.5	2.6
PAI + *c*PAN → D	−16.4	−0.9

**Table 4:** Contact angles of the membranes at 25 °C.

Liquid	Contact Angle, °			
	PAI-0	PAI-5	PAI-10	PAI-15
Water	56.0	35.6	33.3	32.6
Ethylene glycol	33.5	29.5	28.3	24.8

## Data Availability

Data is contained within the article.

## Supplementary Materials

The following supporting information can be downloaded at: https://www.mdpi.com/article/10.3390/membranes12050489/s1; Table S1: Calculated enthalpies, entropies, and Gibbs free energies (in Hartree) for optimized equilibrium model structures (H, S, and G, respectively).
