# Supplementary material for: Application of Cyclized Polyacrylonitrile for Ultrafiltration Membrane Fouling Mitigation
Source: Membranes (Basel). 2022 Apr 30;12(5):489. doi: 10.3390/membranes12050489 (PMC9143852; doi:10.3390/membranes12050489)
Supplement: Supplementary file 1 [file membranes-12-00489-s001.zip › membranes-1695549-supplementary.pdf]

membranes

Supplementary Materials

Application of Cyclized Polyacrylonitrile for Ultrafiltration Membrane Fouling Mitigation

Alexandra Pulyalina [1,*], Nadezhda Tian [1], Anna Senchukova [1], Ilya Faykov [1], Maria Ryabikova [1], Alexander Novikov [1], Natalia Saprykina [2] and Galina Polotskaya [1,2]

[1] Institute of Chemistry, Saint Petersburg State University, Saint Petersburg 198504, Russia; tyan-nadezhda91@yandex.ru (N.T.); st024726@student.spbu.ru (A.S.); st022544@student.spbu.ru (I.F.); st069152@student.spbu.ru (M.R.); a.s.novikov@spbu.ru (A.N.); polotskaya@hq.macro.ru (G.P.)

[2] Institute of Macromolecular Compounds, Russian Academy of Sciences, Saint Petersburg 199004, Russia; saprykina@hq.macro.ru

* Correspondence: a.pulyalina@spbu.ru; Tel.: +78-124-284-805

Table S1. Calculated enthalpies, entropies, and Gibbs free energies (in Hartree) for optimized equilibrium model structures (H, S, and G, respectively).

Model Structure	H	G	S
PAI	0.319999	0.218247	214.155
m-PAN	0.445113	0.371262	155.434
A	0.752333	0.594360	332.484
B	0.751947	0.598635	322.672
C	0.748442	0.593602	325.889
D	0.738990	0.588033	317.716
